# Nasal prosthesis with magnetically secured intranasal framework for a patient with partial rhinectomy and intraoral defects: A case report

**Published:** 2020-10-14

**Authors:** Anjana Kurien, Megashyam Poundass, Subha Anirudhan, Thirumurthy Ramasamy Velliangattur, Bindhoo Arakonam Yuvaraja, Arun Masilamani

**Affiliations:** ^1^Department of Prosthodontics, Sri Ramakrishna Dental College and Hospital, Coimbatore, Tamil Nadu, India; ^2^Newgen Multispeciality Dental and Implant Care, Chennai, Tamil Nadu, India; ^3^Department of Conservative Dentistry and Endodontics, Sri Ramakrishna Dental College and Hospital, Coimbatore, Tamil Nadu, India; ^4^RR Dental Clinic, Coimbatore, Tamil Nadu, India; ^5^Burma Hospital, Coimbatore, Tamil Nadu, India

**Keywords:** squamous cell carcinoma, partial rhinectomy, modified syringe tube impression technique, magnet retained prosthesis, customized intranasal framework

## Abstract

Partial rhinectomy is a challenge for most maxillofacial prosthodontists, as retention of the prosthesis is a major issue to overcome. The scenario becomes even more difficult when there is an intraoral defect. This article describes the fabrication of a removable silicone nasal prosthesis and removable PMMA intraoral obturator prosthesis to rehabilitate a patient following partial rhinectomy with associated loss of premaxilla. The retention of removable intraoral and extraoral prosthesis was achieved partially from anatomical undercuts and further enhanced by rare earth magnets placed between both prostheses. An acrylic framework was fabricated and placed inside to provide rigidity to the nasal part of the prosthesis. A customized method of making impressions using a modified syringe tube to mold the inner surface of the nasal defect is described. The obturator function scale (OFS) questionnaire was used to measure the patient’s satisfaction with the obturator. After insertion of the prosthesis, there was an overall improvement in the OFS of the patient.

**Relevance for patients:**

This case report documents the rehabilitation of a patient following partial rhinectomy and associated loss of premaxilla with customized intraoral obturator prosthesis and a removable silicone nasal prosthesis. The successful outcome of this case shows that for people with similar orofacial defects, these prostheses are a good option to achieve acceptable esthetics, speech, and function.

## 1. Introduction

Maxillofacial defects are acquired due to malignant disease, trauma, or congenital defects. Head and neck cancer surgeries often involve extensive dissection of tissues surrounding the primary lesion. Accompanying facial disfigurement creates a profound negative psychological impact on a person, impairment of speech, and difficulty in deglutition, all of which affect the quality of life and social behavior of the patient [1,2]. Plastic surgery and reconstruction flaps are expensive and by themselves alone cannot restore the functional and esthetic requirements of these patients as much as a maxillofacial prosthesis [3]. Such defects require intricate prosthetic treatment due to the associated aesthetic and psychological problems. Midfacial defects are defined as those confined to the middle third of the face in the horizontal plane and that communicate with intraoral maxillary defects [3]. Marunick *et al*. classified mid-facial defects into two main categories: Midline defect that includes the nose and may also include the upper lip and lateral defects, which include the orbit and cheek [4]. The severity of a defect is consistent with the extent of tissue loss, the relationship between the defect and adjacent structures and cavities such as the brain and oral cavity. Functional challenges such as difficulty in speech, deglutition, control of saliva, and mastication may accompany the usual facial disfigurement and lead to social isolation, loss of employment, and decreased quality of life [5,6].

Maxillofacial prosthodontists can rehabilitate these patients using prosthetic restorations to achieve esthetics and function, where surgical reconstruction is complicated [7]. The aims of prosthetic management in these clinical situations are achieving adequate prosthesis retention, satisfying aesthetics by striving to replicate contralateral side, and merging the prosthesis tissue junction imperceptibly. The use of anatomic undercuts, magnets, implants, and tissue adhesives is some methods of maxillofacial retention. Whenever satisfactory anatomic undercuts are not available, implants are liable method of retention. Over the last five decades, osseointegrated implants have been used to improve the retention of facial prosthesis [8]. However, certain factors can still preclude implant surgery, such as radiation therapy, anatomic complexity, recurring lesions, and the complexity of the procedure. An implant retained nasal prosthesis with implants placed in the floor of the nasal cavity is a good choice for retentive treatment, but data regarding success rate of implants inserted post-radiotherapy remains unclear in the literature [9,10].

In the absence of adequate bone to support implant placement, other retentive options should be analyzed. Magnetic attachments placed on suitable abutment teeth or between portions of sectional prosthesis is another retentive option. This allows comfortable wearing of a removable maxillofacial prosthesis allowing the patient to go about life without drawing attention to their facial deformities [10]. Newer generation rare earth magnets are available in smaller dimensions that can successfully retain intraoral and extraoral prostheses [11-14]. These magnets provide a certain degree of prosthesis movement which is desirable to reduce tissue friction.

The main focus in rehabilitating maxillofacial defects is improving the quality of life of the patient. Many researchers have reported that obturator prosthesis with enhanced retention can improve oral functions post-operatively. Irish *et al*. showed that obturator function scale (OFS) was a useful tool in measuring quality of life and the patients’ response to treatment with maxillofacial prosthesis [15]. The questionnaires have been validated and used by other investigators [15]. The OFS questionnaire has eight domains that include satisfaction with facial appearance, ability to speak in public, leakage of liquids and solids, dryness of mouth, insertion of an obturator, chewing or eating, and social family interactions. Numerical values from 0 being dissatisfaction to 100 being satisfaction for each response in the questionnaires are recorded from the patient, tabulated, and an average calculated to obtain the overall OFS score [16].

This clinical report describes a patient, who presented with loss of premaxilla and midfacial defect due to the left partial rhinectomy. The defect sites lacked adequate bone to allow implant placement. Hence, the rehabilitation of intraoral defect was done with the removable obturator and extraoral defect with removable silicone prosthesis. The intraoral and extraoral prosthesis retention was achieved partially by utilizing available anatomic undercuts in the resected sites and further enhanced with neodymium intraoral magnets placed on a custom-made acrylic stent between both the prostheses.

## 2. Clinical Report

**Figure 1:**
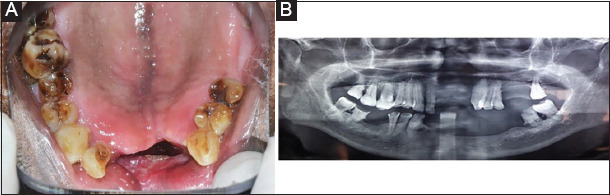
(**A**) Intraoral view of premaxillary defect. (**B**) Orthopantomagram.

**Figure 2:**
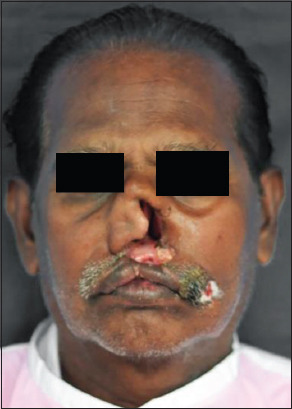
Nasal defect after surgical resection.

A 60-year-old male patient reported to the department of prosthodontics with the complaint of an ill-fitting oronasal prosthesis that had been inserted to restore a partial rhinectomy defect. The patient had undergone left partial rhinectomy for management of squamous cell carcinoma of floor of nose 1 year before his visit. The surgical procedure involved partial resection of the left side of the nose along with the left ala, tip, columella, nasal septum till the vomer bone, and the extraction of teeth 11, 12, and 21 (Figure 1A and B). He had been previously rehabilitated with a silicone nasal prosthesis retained using a spectacle. Ten months after surgery, the patient developed a secondary lesion and a second surgery was carried out to resect around 1 cm from the existing margin till the midline involving the dorsum and tip of nose and teeth 15, 22, 24, 25, 26, and 27 were extracted followed by radiotherapy. The patient requested a replacement of the prosthesis 3 months after surgery as the existing prosthesis was non-retentive with poor color match. Extraoral examination after second surgical procedure showed resection of the nose on the left side along with the septum and obliteration of the left nasolabial fold. A midline incision during surgery had resulted in an everted upper lip (Figure 2). Mouth opening was satisfactory. Intraoral examination revealed orofacial communication and partially edentulous maxillary arch with missing teeth 11, 12, 15, 21, 22, 24, 25, 26, and 27 showing Class 2 brown classification [17]. Periodontal status of remaining teeth was satisfactory for prosthesis retention. Intraoral photograph with cheek retractor was not possible at this stage as there was another painful lesion at the left corner of mouth. In light of the compromised bone quality and quantity in the defect site, and considering the patient’s socioeconomic status, a decision was made to fabricate a removable silicone nasal prosthesis for the extraoral defect that could be magnetically attached to a removable heat-cured PMMA obturator placed intraorally. The prosthesis was planned in two pieces for ease of placement and removal and maintenance hygiene.

### 2.1. Fabrication of the heat-cured PMMA obturator

**Figure 3:**
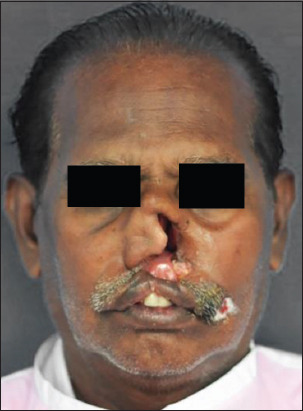
Acrylic removable partial denture with labial flange extending into the defect.

**Figure 4:**
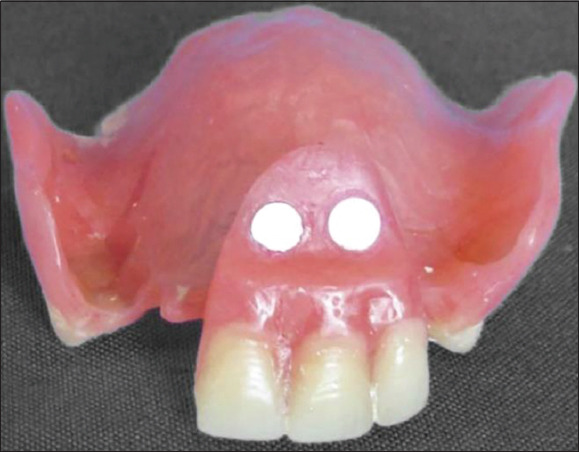
Two neodymium magnets embedded in the labial mold.

The initial prosthetic approach was to fabricate a maxillary acrylic partial denture to replace the missing teeth. The labial flange of the denture was designed to extend vertically until the floor of nasal cavity to engage the nasal communication. After processing, the obturator was inserted into the patient’s mouth (Figure 3) and the midline of vertical extension was marked. Two holes of 4 mm diameter and 2 mm depth were drilled 3 mm from the marked midline. Two neodymium magnets of 3 mm diameter and 1.5 mm height (D 21, K&J Magnetics, Jamison, PA, USA) were secured into these holes using self-cure acrylic (DPI-RR Self Cure, DPI, Mumbai, India) (Figure 4).

### 2.2. Fabrication of the silicone nasal prosthesis

**Figure 5:**
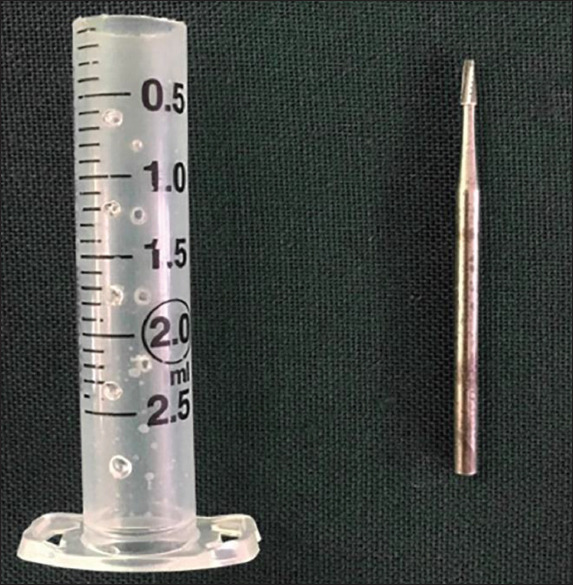
Modified syringe for impression rendering.

**Figure 6:**
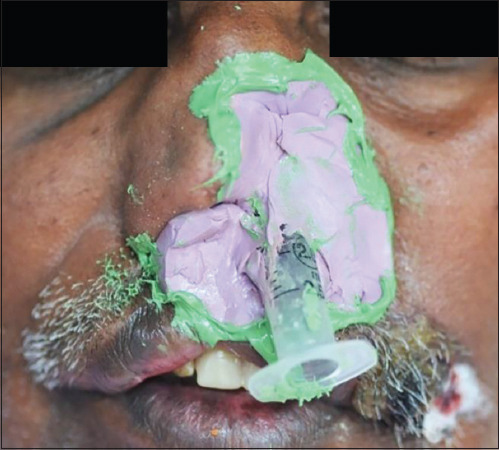
Impression of the defect by using polyvinyl siloxane.

**Figure 7:**
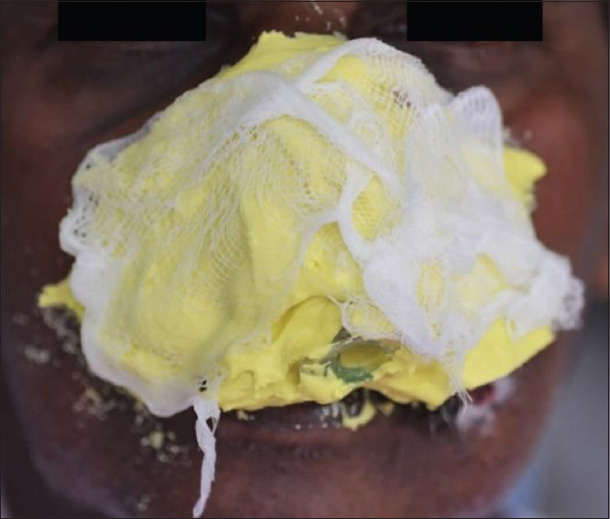
Facial moulage, defect covered with alginate.

**Figure 8:**
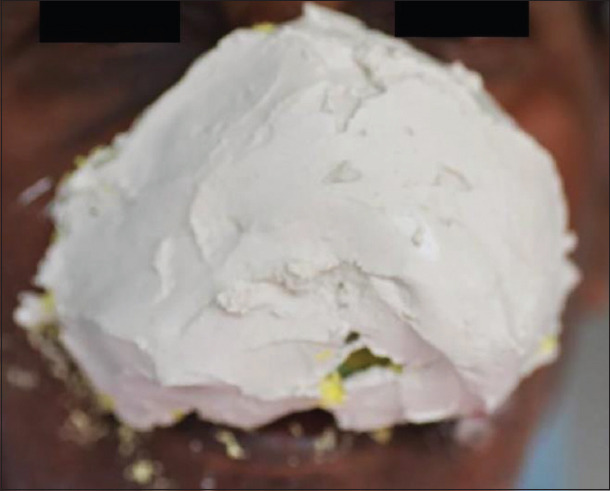
Alginate covered with gauze and plaster.

**Figure 9:**
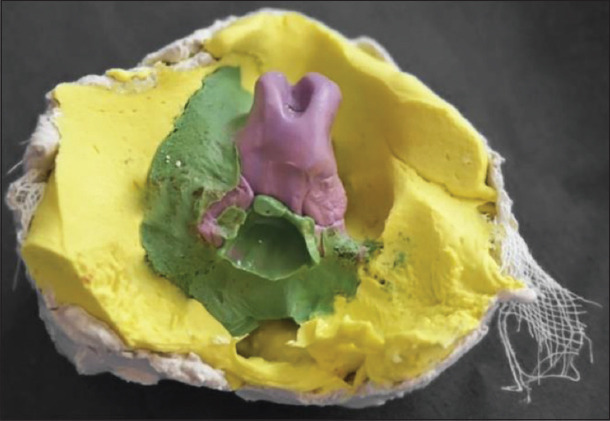
Final impression of the mold.

**Figure 10:**
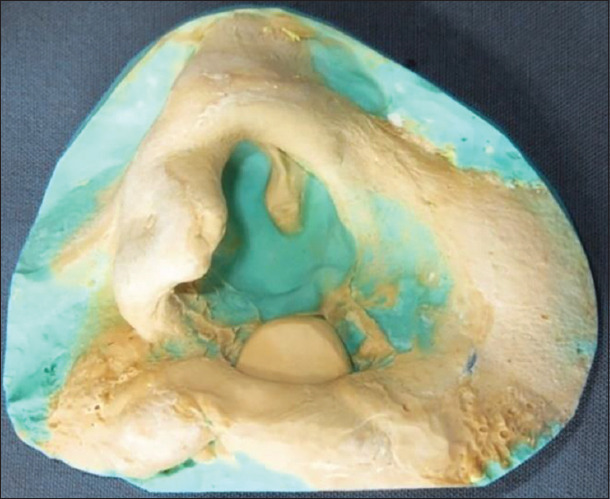
Working cast with denture flange extension.

The first step in the fabrication of a definitive nasal prosthesis was to make an impression of the defect with the obturator in place. A customized impression carrier was prepared by removing the nozzle and hub portion of a disposable 2-mL syringe tube. The disposable syringe barrel was used for this impression rendering as it is readily available, easily modifiable, and transparent, permitting to visualize the flow of impression material (Dispovan, Hindustan Syringes and Medical Devices, Faridabad, Haryana, India). Small retentive holes were drilled through this barrel portion to mechanically retain the set impression on retrieval (Figure 5). Tray adhesive (VPS tray adhesive, 3M ESPE, Seefeld, Germany) was applied on the surface of the tube. Polyvinyl siloxane (PVS) silicone putty (Express, 3M ESPE) was loaded around the cut end of the syringe, carried over to the defect, and molded to obtain the peripheral impression of the defect. Light body PVS (Express, 3M ESPE) was injected through the barrel to record the anatomical undercuts of the defect in a passive state (Figure 6). With the PVS impression in place, the defect and surrounding middle third of the face was recorded with low viscosity alginate (Tropicalgin, Zhermack, Badia Polesine, Italy) (Figure 7). Gauze and plaster were packed over it to obtain a facial moulage (Figures 8 and 9). The final impression was disinfected and a working cast was poured using Type 4 dental plaster (FUJIROCK, GC International, Luzern, Switzerland) (Figure 10).

**Figure 11:**
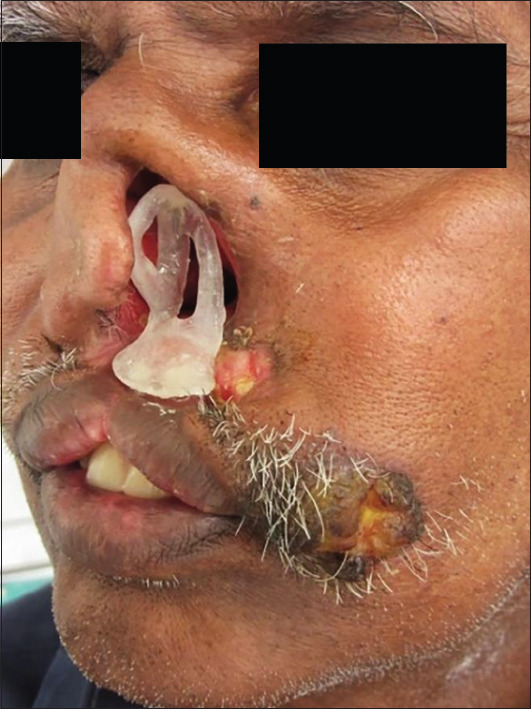
Acrylic framework in position.

**Figure 12:**
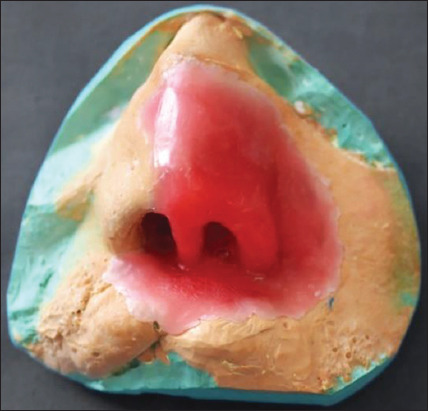
Wax pattern on working cast

**Figure 13:**
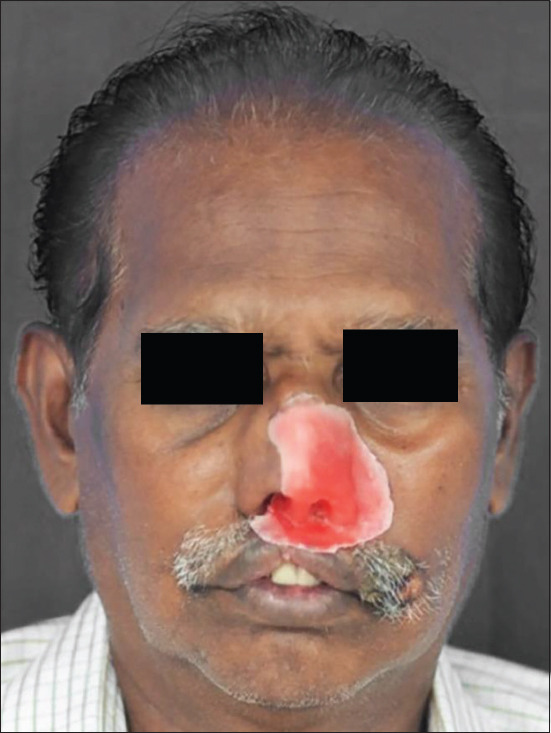
Wax pattern in situ.

**Figure 14:**
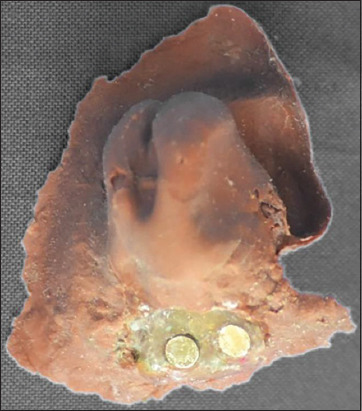
Magnets in framework opposing the labial flange of denture.

**Figure 15:**
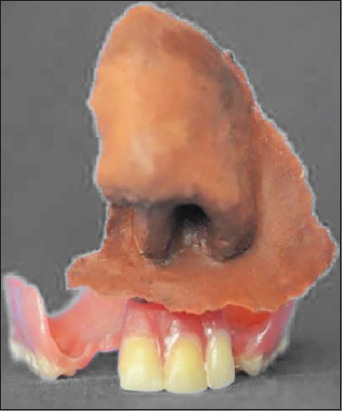
Magnetically attached intraoral and extraoral prosthesis.

**Figure 16:**
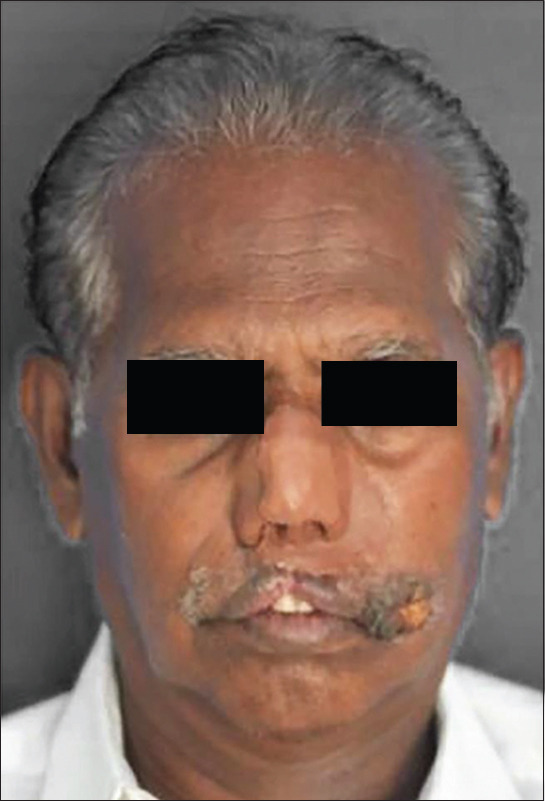
Final prosthesis in place.

**Figure 17:**
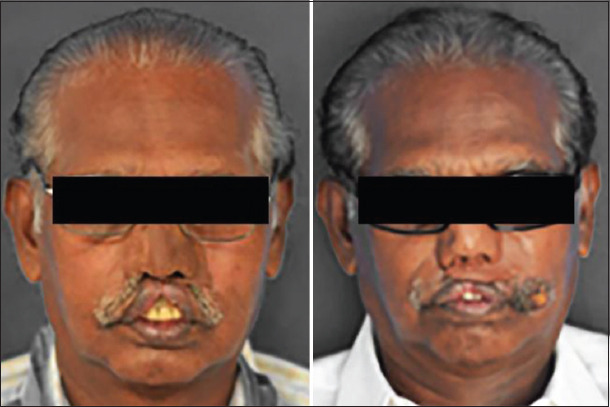
Before and after comparison.

On the working cast, a heat-cured PMMA framework was fabricated. The framework had an oral portion spread over the vertically extended labial flange of the obturator and a nasal portion extending vertically over the septum of the nose and over the lateral resected portion of the nose. Small through-and-through slots were drilled on the stent to allow airway passage. The framework was to be embedded in the silicone nasal prosthesis. It served to engage anatomical undercuts, helped to retain magnets, and improved strength and rigidity of silicone nasal prosthesis. Once the processed framework was verified on the patient (Figure 11), the framework was stabilized on the working cast and a wax pattern for the nasal prosthesis was carved using hard wax (MAARC hard modeling wax, Shiva Products, Maharashtra, India). It was tried on the patient and the fit and symmetry were evaluated (Figures 12 and 13). The waxed-up nasal prosthesis, along with the framework, was flasked, dewaxed, and the patency of the nasal airways was blocked with plaster. The mold was painted with separating medium. Shade matching pigments (skin shades: soft brownF1-SK15 and cream F1-SK07; ahesives B230 Daro Adhesives Hydrobond; Technovent, South Wales, U.K.) were added to RTV silicone (silicone M51 addition platinum silicone rubber, Technovent, South Wales, U.K.) and packed into the mold. The silicone was cured for 48 h, removed from the flask, trimmed, and tried on the patient. The position of the magnets corresponding to that on the obturator flange was marked and drilled on the underside of the nasal prosthesis over the acrylic framework. The counterpart magnets were placed over the obturator flange and were picked up by the nasal prosthesis (Figures 14 and 15) with the help of self-cure PMMA resin. The completed prosthesis was then inserted (Figure 16). The patient was advised to place the intraoral component first and then attach the extraoral nasal prosthesis. Instructions regarding hygiene and maintenance of silicone prosthesis were given. The patient was followed-up and examined for fit and comfort of prosthesis after 1 week. Regular check-up was done every 2 months. An OFS comprising questionnaire related to esthetics, function, phonetics, social interaction, and patient comfort was measured for the patient. The OFS of the patient was measured initially with the old prosthesis and with the new prosthesis after 2 weeks of insertion. Numerical value from 0 to 100 for each response in the questionnaire was scored by the patient and the average calculated. There was a marked improvement in the OFS scores for the patient from the older prosthesis of 54 to the newer magnet retained prosthesis of 66 (Figure 17). Parameters that showed improvement were satisfaction with facial appearance, speech, swallowing, leakage with liquids, insertion of the obturator, and social and family interaction.

## 3. Discussion

The prosthodontist managing combined facial and intraoral defects is faced with the dual challenge of not only rehabilitating the facial defects but also to restore the function of speech, mastication, and deglutition. Securing the prosthesis in place can be a formidable task due to its size and weight [14,18].

Among the choices of prosthesis retention are anatomic undercuts, implants, magnets, tissue adhesives, eyeglass retention, or a combination thereof. The adhesive usage requires patients to apply the adhesive to the prosthesis periphery at each time of usage. Some patients are uncomfortable with the mucin-like appearance of adhesives, and some even develop an allergic reaction to the adhesive. As for eye glass retention, patients who do not wear glasses regularly may not prefer the option of having to wear one. Implants which are osseointegrated provide the most reliable retention for these prostheses. However, additional surgeries, expenses, inadequate bone, and prior radiation to this area do not favor such an approach. A magnetic attachment placed on remaining dentition is another treatment of choice when implants are not feasible. However, loss of retentive force with time is an issue [19,20].

This case report discusses the fabrication sequence of a silicone nasal prosthesis and heat-cured PMMA maxillary obturator prosthesis for a combined midfacial and premaxilla defect. The patient was an old prosthesis wearer who needed a remake following second surgery due to cancer relapse. The main concern of the patient was the need for better retention, unlike his old prosthesis which was retained by eyeglasses. After evaluation of the defect site, which presented inadequate bone to support the implant, an alternate approach was considered for prosthesis retention. As the patient presented with intra- and extra-oral defects, the prosthesis was partially retained with respective anatomic undercuts and additional rare earth magnetic attachments were placed between the intraoral and extraoral prosthesis to enhance mutual retention [13]. A custom-made acrylic framework was incorporated into the nasal prosthesis to secure the magnets and also to improve the rigidity to the prosthesis.

Although 3D planning and 3D printing constitute more advanced methods of generating prostheses, which would have given a more accurate anatomic protype with excellent facial symmetry [21], conventional wax pattern was generated on the master cast due to economical constraints of the patient.

Heat-cured PMMA obturator and silicone nasal prostheses were fabricated. The material selection for the intraoral prosthesis and extraoral prosthesis were selected keeping in mind the functional demands and esthetic requirements of the respective prosthesis. The average functional lifespan of a silicone nasal prosthesis is reported to be 1.5–2 years [19]. The functional lifespan of a PMMA obturator can be as long as 4–5 years [22,23]. The intraoral obturator can act as a base, for recording the intaglio surface of the nasal prosthesis during its fabrication.

The two-piece design allowed the patient to remove the prosthesis, clean it, and replace it with ease. During the revisit, it was observed that the patient maintained good hygiene of the oral cavity and the nasal prosthesis. Furthermore, the patient had improved esthetics and there was a reduction in hyper nasal speech. The patient reported that his self-esteem had improved and his relatives reported that he was socializing more. The patient was satisfied with the enhanced retention. Rogers *et al.*, in his study, said that patients with larger defects had lower scores for activity, recreation, and physical function compared to those with smaller defects [24].

Irish *et al*. [15] showed that OFS score is a useful tool in measuring the quality of life in patients with the maxillofacial prosthesis. A cohort study conducted by Chen *et al*. [16] reported that retention was enhanced in prosthesis by the addition of an attachment and also showed more benefits in oral functions. There was a marked improvement in the OFS scores for this patient from the older prosthesis of 54.44 to the newer magnet-retained prosthesis of 66.11.

## 4. Summary

This case report highlights the important aspects in the treatment planning for a midfacial defect patient, in which implant treatment was not an option due to poor bone volume in the defect site. Further, technical difficulties such as undercuts and a poor dentition had to be overcome to rehabilitate the patient. The difficulties of the patient were kept in mind and appropriate fabrication method was selected. Removable heat cure PMMA obturator was the intra oral prosthesis to replace lost dentition and to improve function. Removable silicone nasal prosthesis was fabricated to restore the midfacial defect. Rare earth neodymium magnets with reasonable retentive force were chosen for mutual retention of intra- and extra-oral prosthesis. The final two-piece prosthesis greatly enhanced the OFS score of the patient.
